# Supplementary material for: Using early detection data to estimate the date of emergence of an epidemic outbreak
Source: PLoS Comput Biol. 2024 Mar 8;20(3):e1011934. doi: 10.1371/journal.pcbi.1011934 (PMC10954163; doi:10.1371/journal.pcbi.1011934)
Supplement: S1 Text — Fig A. Reanalysis of Roberts et al. (2021) [4] with updated datasets. The original analysis was done with Huang et al.’s dataset [5]. We re-ran the analysis on updated case datasets, using the same N = 10 number of case-days as in the original analysis. Fig B. Distribution of the difference (in days) between the estimated date of the first infection and the actual date of the first infection in simulated data, for different values of N, the number of cases considered for the evaluation. The vertical lines represent the median (full line) and the mean (dashed line) of the distributions. The different simulated datasets are gathered together. Fig C. Estimated dates of first infection, for each of the 100 simulated datasets. The dates of first infection in the simulated datasets are set at 0. The squares show the dates at which there were N = 1, 10, 1000 cases in the simulated dataset, while the diamond, cross and bars show the mean, median and 95% interval of estimated dates of the first infection corresponding to each N, using the simulated dataset as source data. A perfect estimation lands on 0. Fig D. Alpha sequences in the UK. We apply our model to 406 sequenced samples (blue) collected up to November 11 (light-blue highlight) and reported by November 30, 2020 (date of submission to GISAID [36]). We do not consider samples collected between November 12 and November 30 to avoid the effects of reporting delays in the last days. For comparison, here we present the sequences reported after November 30, and up to December 15 (white), corresponding to a total of 455 sequenced samples. Fig E. Cumulative cases of Alpha variant in the UK. Accepted simulations (gray) and observed data (black). Fig F. Estimates of the emergence date using information on the first case only. Distributions of the emergence date for the Alpha variant in the UK, estimated using data on the first observed Alpha case (i.e., September 20, 2020) only, using our model (violet, upper middle) and the m [file pcbi.1011934.s001.pdf]

Appendix

Using early detection data to estimate the date of emergence of an epidemic outbreak

Sofía Jijón[1*], Peter Czuppon[2], François Blanquart[3] and Florence Débarre[1†]

[1]Institute of ecology and environmental sciences of Paris (iEES-Paris, UMR 7618), Sorbonne Université, CNRS, UPEC, IRD, INRAE, Paris, France
[2]Institute for Evolution and Biodiversity, University of Münster, Münster, Germany
[3]Center for Interdisciplinary Research in Biology, CNRS, Collège de France, PSL Research University, Paris, France

[*]Corresponding author. E-mail: sofia.jijon@gmail.com
[†]Corresponding author. E-mail: florence.debarre@normalesup.org

Contents

1 Reanalysis of Roberts et al. (2021) 4

2 Running our model on simulated data 5

3 Supplementary tables 7

4 Alpha SARS-CoV-2 variant in the UK 11

4.1 Alpha sequences reported in the UK 11

4.2 GISAID data on Alpha cases in the UK 12

4.3 Cumulative cases 13

4.4 Using the first Alpha case only 14

5 COVID-19 in Wuhan 15

5.1 Early cases of COVID-19 reported in Wuhan 15

5.2 Cumulative cases 16

5.3 Using other COVID-19 datasets 17

6 Pseudo-algorithm 19

References 21

List of supplementary figures

A Reanalysis of Roberts et al. (2021) [1] with updated datasets 4

B Distribution of the difference (in days) between the estimated date of the first infection and the actual date of the first infection in simulated data 5

C Estimated dates of first infection, for each of the 100 simulated datasets 6

D Alpha sequences in the UK 11

E Cumulative cases of Alpha variant in the UK 13

F Estimates of the emergence date using information on the first case only 14

G Early COVID-19 cases in Wuhan 15

H Cumulative cases of COVID-19 in Wuhan 16

I Estimates of the emergence date using truncated or outdated case datasets 17

List of supplementary tables

A Model calibration 7

B Estimates for the emergence date 8

C Sensitivity analyses 9

D Impact of using different datasets 18

1 Reanalysis of Roberts et al. (2021)

We used the same methodology as Roberts et al. (2021) [1] and ran it on updated datasets to assess the consequences of changes in the input case data. We found that the most recent datasets led to much later estimated dates of the first infection (see Figure A).

Figure A: Reanalysis of Roberts et al. (2021) [1] with updated datasets. The original analysis was done with Huang et al.'s dataset [2]. We re-ran the analysis on updated case datasets, using the same $N = 10$ number of case-days as in the original analysis.

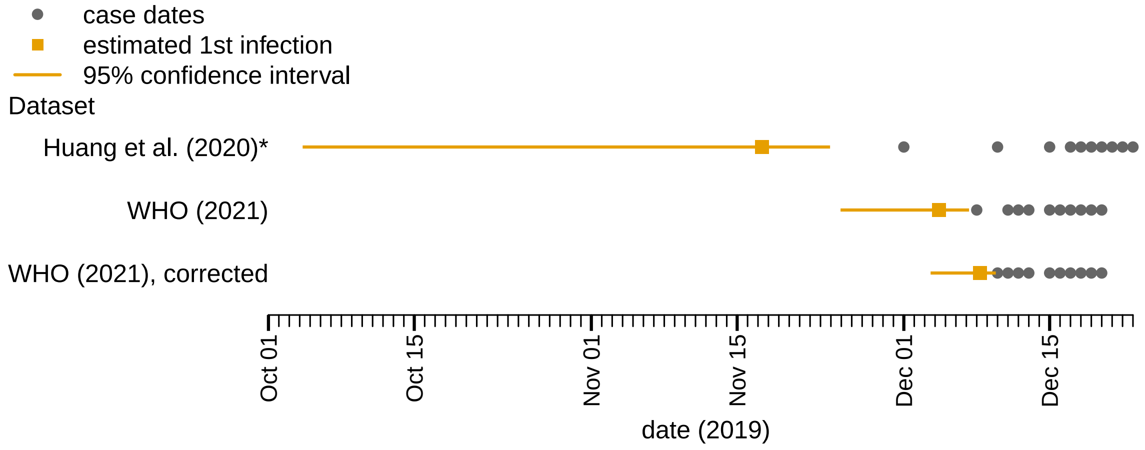

2 Running our model on simulated data

Here, we ran our model on simulated case datasets and quantified the error of our estimates versus the *observed* date of emergence (observed in the simulation scenario and thus known). We generated 100 simulated datasets and further ran our model, with 1000 replicates for each combination, on subsets of different sizes; that is, we considered the case data up to the day of occurrence of the Nth case, for increasing values of $N =\sim 1$, $N =\sim 10$ and $N =\sim 1\,000$. Overall, the estimated date of first infection is slightly earlier that the real date of first infection (Figure B).

Figure B: Distribution of the difference (in days) between the estimated date of the first infection and the actual date of the first infection in simulated data, for different values of N, the number of cases considered for the evaluation. The vertical lines represent the median (full line) and the mean (dashed line) of the distributions. The different simulated datasets are gathered together.

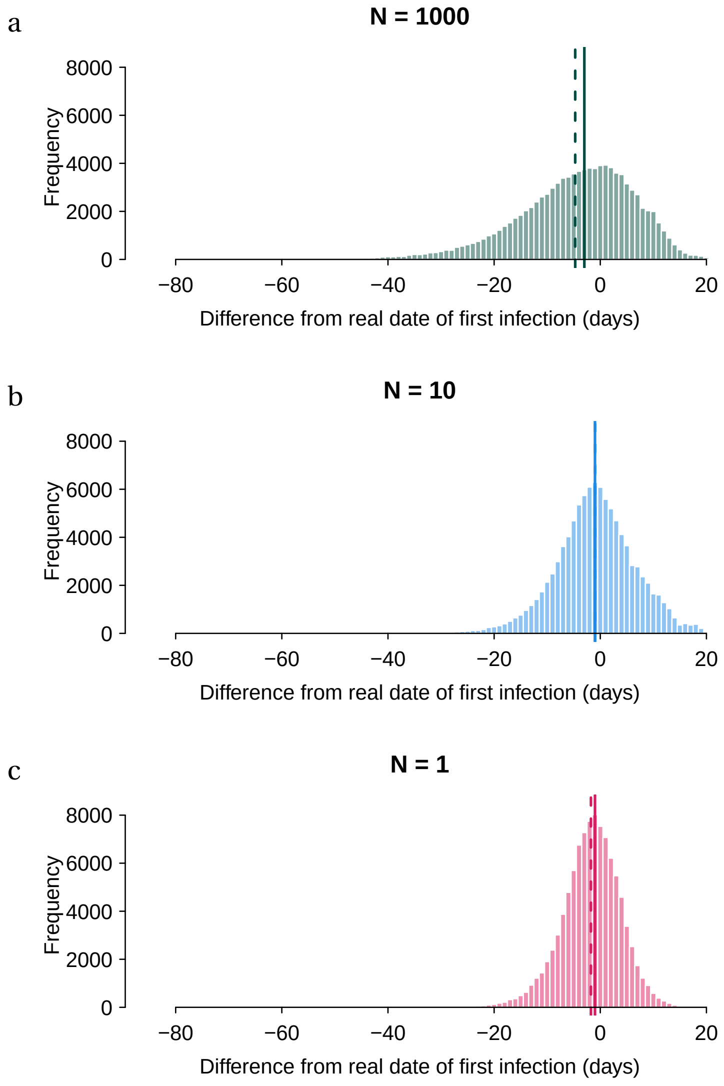

We also explored whether the real date of first infection was within the 95% IPR for each simulation. When combining estimations with $N = 1$ and $N = 1000$, all simulations had the real first infection date in the 95% IPR. With individual values of N, the first date of infection could occasionally be outside of the 95% IPR, the estimation being mostly at an earlier date than the simulated data's date (Figure C).

Figure C: Estimated dates of first infection, for each of the 100 simulated datasets. The dates of first infection in the simulated datasets are set at 0. The squares show the dates at which there were $N = 1$, 10, 1000 cases in the simulated dataset, while the diamond, cross and bars show the mean, median and 95% interval of estimated dates of the first infection corresponding to each N, using the simulated dataset as source data. A perfect estimation lands on 0.

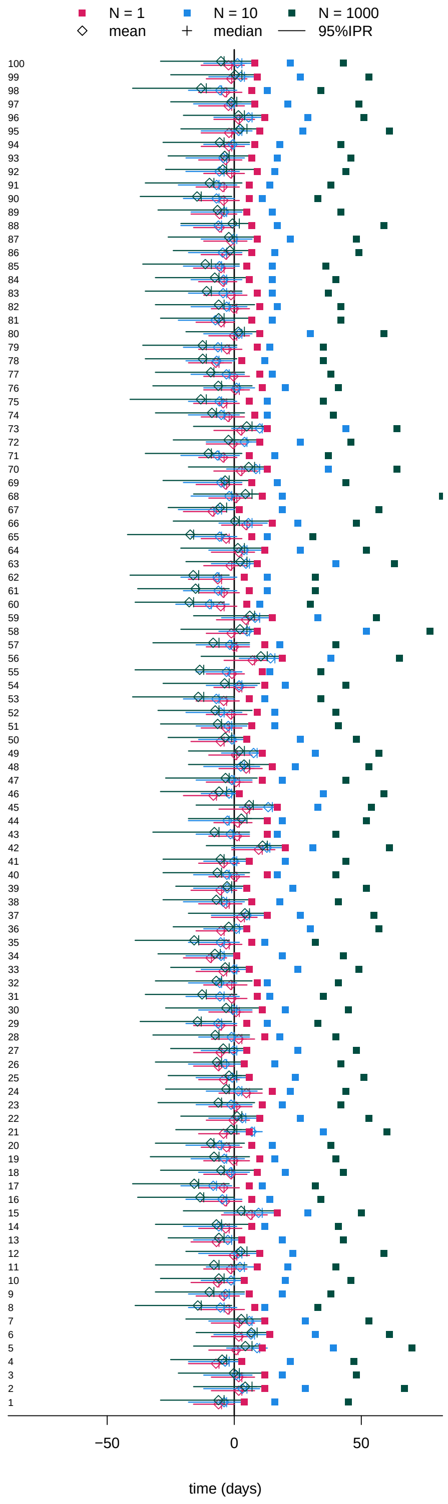

3 Supplementary tables

Table A: Model calibration. Our results are obtained from a set of 5 000 simulations selected by the model calibration. Here, we summarize some metrics obtained by calibrating our model for the two applications, and show the observed data for comparison. The values and ranges of estimates correspond to medians and 95% interpercentile ranges.

	**Alpha**		**COVID-19**	
**Metric**	**Observed**	**Estimated**	**Observed**	**Estimated**
Date of 1st case	Sep 20	Sep 21 (Aug 24–Oct 4)	Dec 10	Dec 8 (Nov 13–Dec 18)
Delay from 1st to N^{th} case (days)	53	51 (38–79)	41	42 (32–67)
Total cases at day of Nth case	406	432 (407–465)	3072	~3 360 (3 080–3 690)
Proportion of accepted simulations	–	~ 36%	–	~ 14%

Table B: Estimates for the emergence date. Estimated dates of emergence. Median values and central 95% interpercentile ranges (values between 2.5^{th} and the 97.5^{th} percentiles) are shown. Abbreviations: tMRCA= time of most recent ancestor.
* Simulations run using the model presented in [3], but with updated parameters matching ours.

	Method	Date of emergence	Earliest infection	Study
**Alpha**	Population dynamics, based on N observations.	Aug 21 (Jul 23–Sep 5), 2020	Jun 13, 2020	This study
	Population dynamics, based on the first observation.	Aug 19 (Jul 19–Sep 9), 2020	Jun 2, 2020	[3] updated*
	Phylodynamics estimating tMRCA.	Aug 28 (Aug 14–Sep 9), 2020	Aug 6, 2020	[4]
**COVID-19**	Population dynamics, based on N observations.	Nov 28 (Nov 2–Dec 9), 2019	Sep 26, 2019	This study
	Phylodynamics model coupling transmission and viral genome evolution.	Nov 18 (Oct 16–Dec 6), 2019	Sept 13, 2019	[5]

Table C: Sensitivity analyses. Estimated dates of emergence, obtained by varying the key epidemiological parameters (reproduction number, over-spreading and detection) as well as the parameters regarding the model calibration (i.e., the tolerances for simulation selection; see details in the Methods section of the main text). Median values and 95% interpercentile ranges (values between 2.5th and the 97.5th percentiles) are shown. Baseline values are marked in boldface.

	R	κ	p_{detect}	θ_{τ}	δ^{Y}_{tol}	Date of emergence
**Alpha**	1.7	0.57	0.0105	12	0.3	Aug 8 (Jul 6–Aug 26), 2020
	**1.9**	**0.57**	**0.0105**	**12**	**0.3**	**Aug 21 (Jul 23–Sep 5), 2020**
	2.1	0.57	0.0105	12	0.3	Aug 29 (Aug 3–Sep 12), 2020
	1.9	0.35	0.0105	12	0.3	Aug 23 (Jul 21–Sep 8), 2020
	**1.9**	**0.57**	**0.0105**	**12**	**0.3**	**Aug 21 (Jul 23–Sep 5), 2020**
	1.9	0.75	0.0105	12	0.3	Aug 20 (Jul 18–Sep 3), 2020
	1.9	0.57	0.0050	12	0.3	Aug 14 (Jul 18–Aug 29), 2020
	**1.9**	**0.57**	**0.0105**	**12**	**0.3**	**Aug 21 (Jul 23–Sep 5), 2020**
	1.9	0.57	0.0250	12	0.3	Aug 28 (Jul 31–Sep 11), 2020
	1.9	0.57	0.0105	7	0.3	Aug 23 (Jul 27–Sep 6), 2020
	**1.9**	**0.57**	**0.0105**	**12**	**0.3**	**Aug 21 (Jul 23–Sep 5), 2020**
	1.9	0.57	0.0105	15	0.3	Aug 19 (Jul 23–Sep 2), 2020
	**1.9**	**0.57**	**0.0105**	**12**	**0.3**	**Aug 21 (Jul 23–Sep 5), 2020**
	1.9	0.57	0.0105	12	0.5	Aug 20 (Jul 24–Sep 4), 2020
**COVID-19**	2.0	0.10	0.1500	6	0.3	Nov 17 (Oct 20–Dec 2), 2019
	**2.5**	**0.10**	**0.1500**	**6**	**0.3**	**Nov 28 (Nov 2–Dec 9), 2019**
	3.5	0.10	0.1500	6	0.3	Dec 5 (Nov 14–Dec 10), 2019
	2.5	0.05	0.1500	6	0.3	Nov 30 (Nov 7–Dec 10), 2019
	**2.5**	**0.10**	**0.1500**	**6**	**0.3**	**Nov 28 (Nov 2–Dec 9), 2019**
	2.5	0.25	0.1500	6	0.3	Nov 25 (Oct 29–Dec 5), 2019
	2.5	0.10	0.1000	6	0.3	Nov 26 (Oct 31–Dec 8), 2019
	**2.5**	**0.10**	**0.1500**	**6**	**0.3**	**Nov 28 (Nov 2–Dec 9), 2019**
	2.5	0.10	0.2500	6	0.3	Nov 30 (Nov 4–Dec 10), 2019
	2.5	0.10	0.1500	3	0.3	Dec 1 (Nov 5–Dec 10), 2019
	**2.5**	**0.10**	**0.1500**	**6**	**0.3**	**Nov 28 (Nov 2–Dec 9), 2019**
	2.5	0.10	0.1500	12	0.3	Nov 23 (Oct 28–Dec 5), 2019
	2.5	0.10	0.1500	6	0.1	Nov 28 (Nov 4–Dec 10), 2019
	**2.5**	**0.10**	**0.1500**	**6**	**0.3**	**Nov 28 (Nov 2–Dec 9), 2019**
	2.5	0.10	0.1500	6	0.5	Nov 28 (Nov 2–Dec 10), 2019

4 Alpha SARS-CoV-2 variant in the UK

4.1 Alpha sequences reported in the UK

Figure D: Alpha sequences in the UK. We apply our model to 406 sequenced samples (blue) collected up to November 11 (light-blue highlight) and reported by November 30, 2020 (date of submission to GISAID [6]). We do not consider samples collected between November 12 and November 30 to avoid the effects of reporting delays in the last days. For comparison, here we present the sequences reported after November 30, and up to December 15 (white), corresponding to a total of 455 sequenced samples.

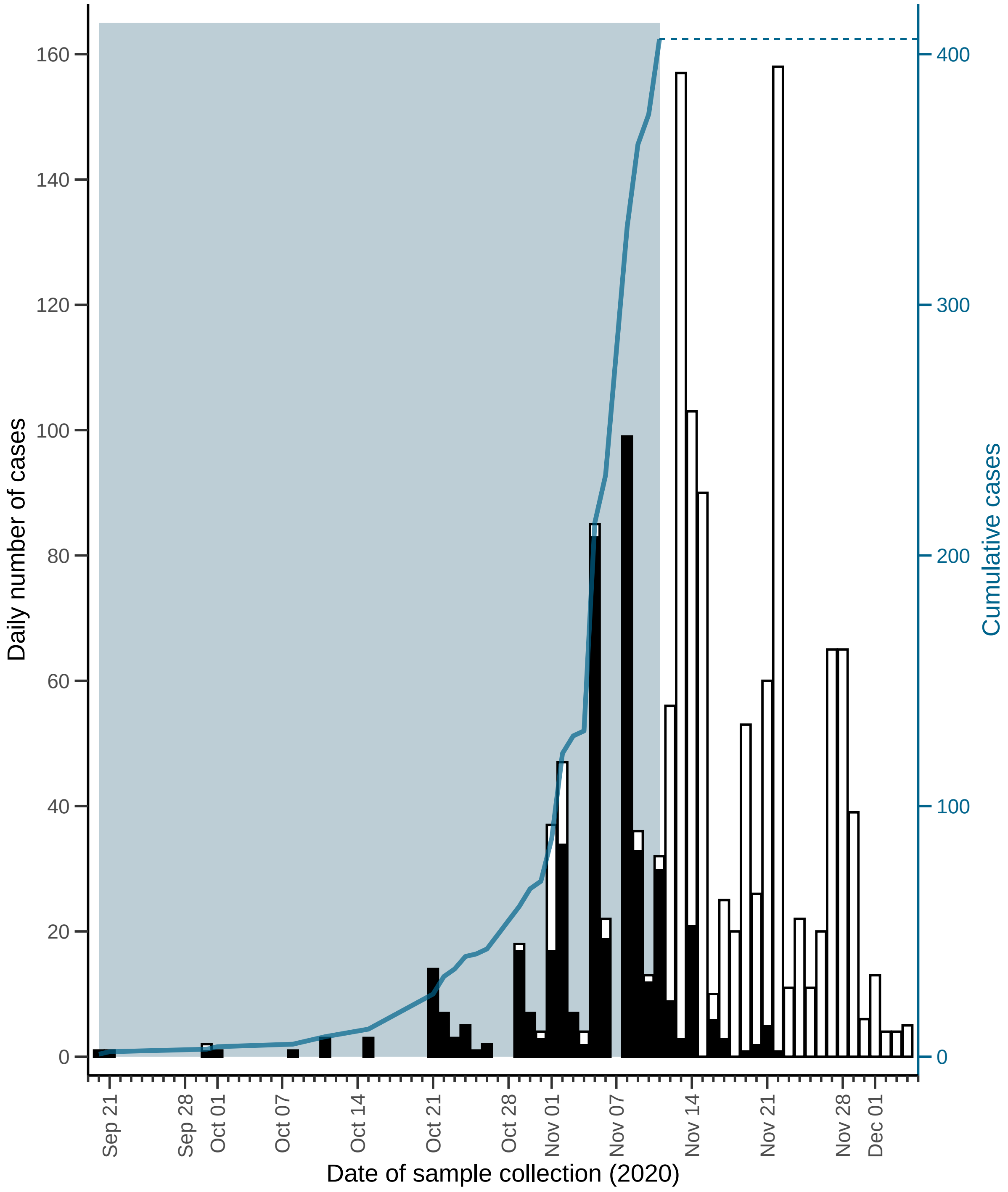

4.2 GISAID data on Alpha cases in the UK

SUPPLEMENTAL TABLE

Data Availability

GISAID Identifier: EPI_SET_230104xg
doi: 10.55876/gis8.230104xg

All genome sequences and associated metadata in this dataset are published in GISAID's EpiCoV database. To view the contributors of each individual sequence with details such as accession number, Virus name, Collection date, Originating Lab and Submitting Lab and the list of Authors, visit 10.55876/gis8.230104xg

Data Snapshot

- EPI_SET_230104xg is composed of 409 individual genome sequences.
- The collection dates range from 2020-09-20 to 2020-11-11;
- Data were collected in 1 countries and territories;
- All sequences in this dataset are compared relative to hCoV-19/Wuhan/WIV04/2019 (WIV04), the official reference sequence employed by GISAID (EPI_ISL_402124). Learn more at https://gisaid.org/WIV04.

4.3 Cumulative cases

Figure E: Cumulative cases of Alpha variant in the UK. Accepted simulations (gray) and observed data (black).

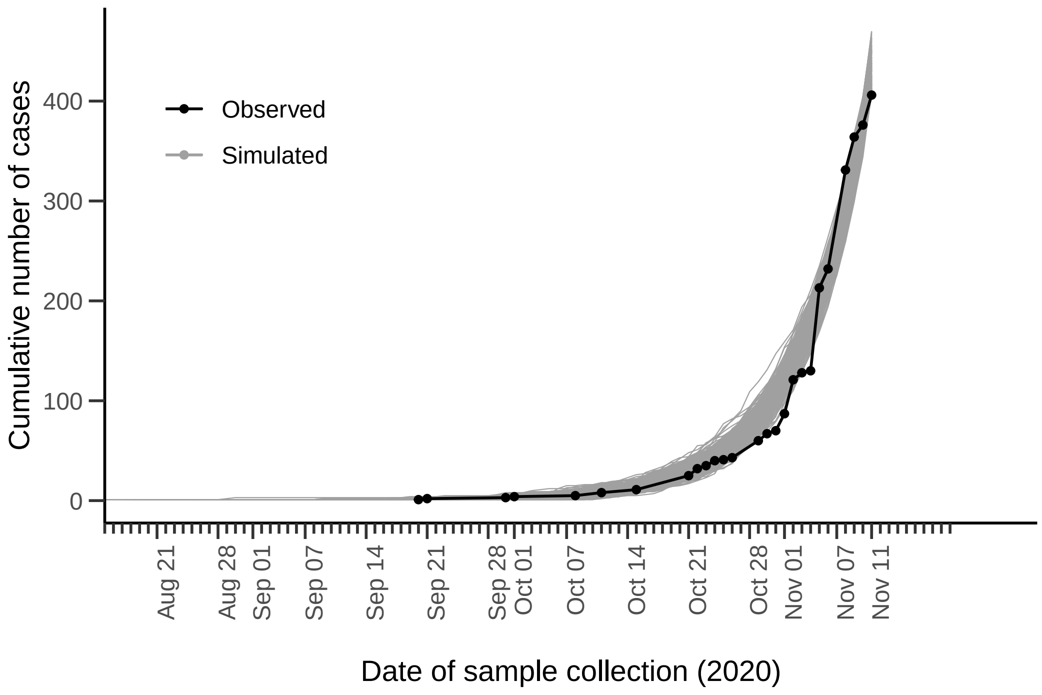

4.4 Using the first Alpha case only

Figure F: Estimates of the emergence date using information on the first case only. Distributions of the emergence date for the Alpha variant in the UK, estimated using data on the first observed Alpha case (i.e., September 20, 2020) only, using our model (violet, upper middle) and the model previously developed by (Czuppon et al., 2021) as published (yellow, bottom) and updating the parameters to match ours (red, lower middle; cf. the Methods section of the main text). We also plot the results from running our model on data of samples collected up to November 11, 2020 (i.e., our main results; blue, top), for comparison.

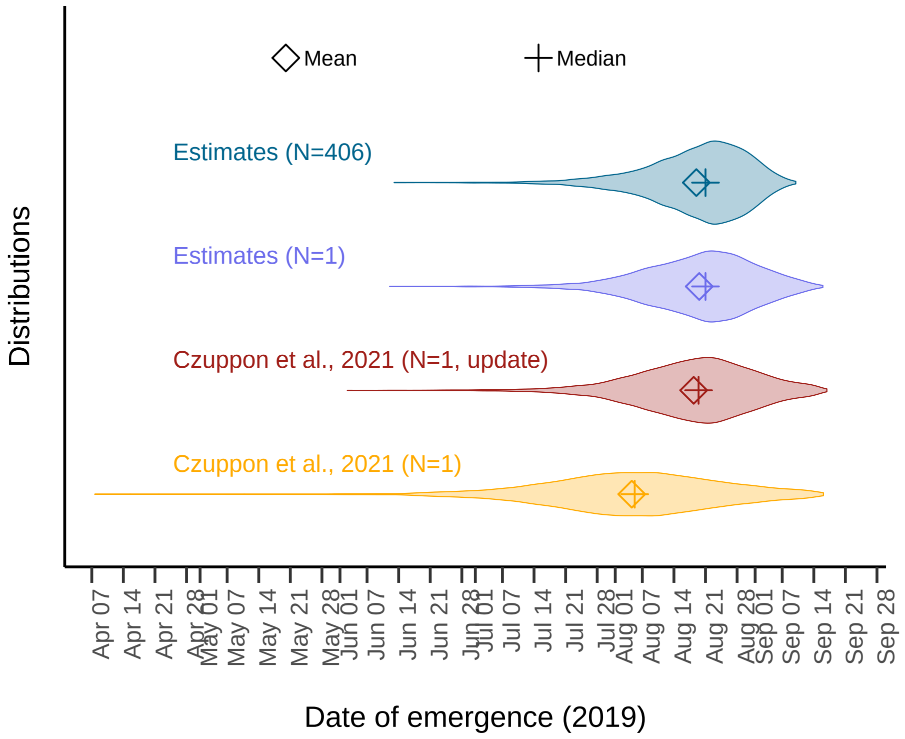

5 COVID-19 in Wuhan

5.1 Early cases of COVID-19 reported in Wuhan

Figure G: Early COVID-19 cases in Wuhan. We apply our model to 3072 COVID-19 reported cases with symptoms onset by January 19, 2020 (blue). On January 20, the first public declaration of human-to-human transmission of the virus was made. Soon after that, the lockdown intervention was deployed nationally, along with testing, which explains the change in epidemic dynamics observed after that date (white).

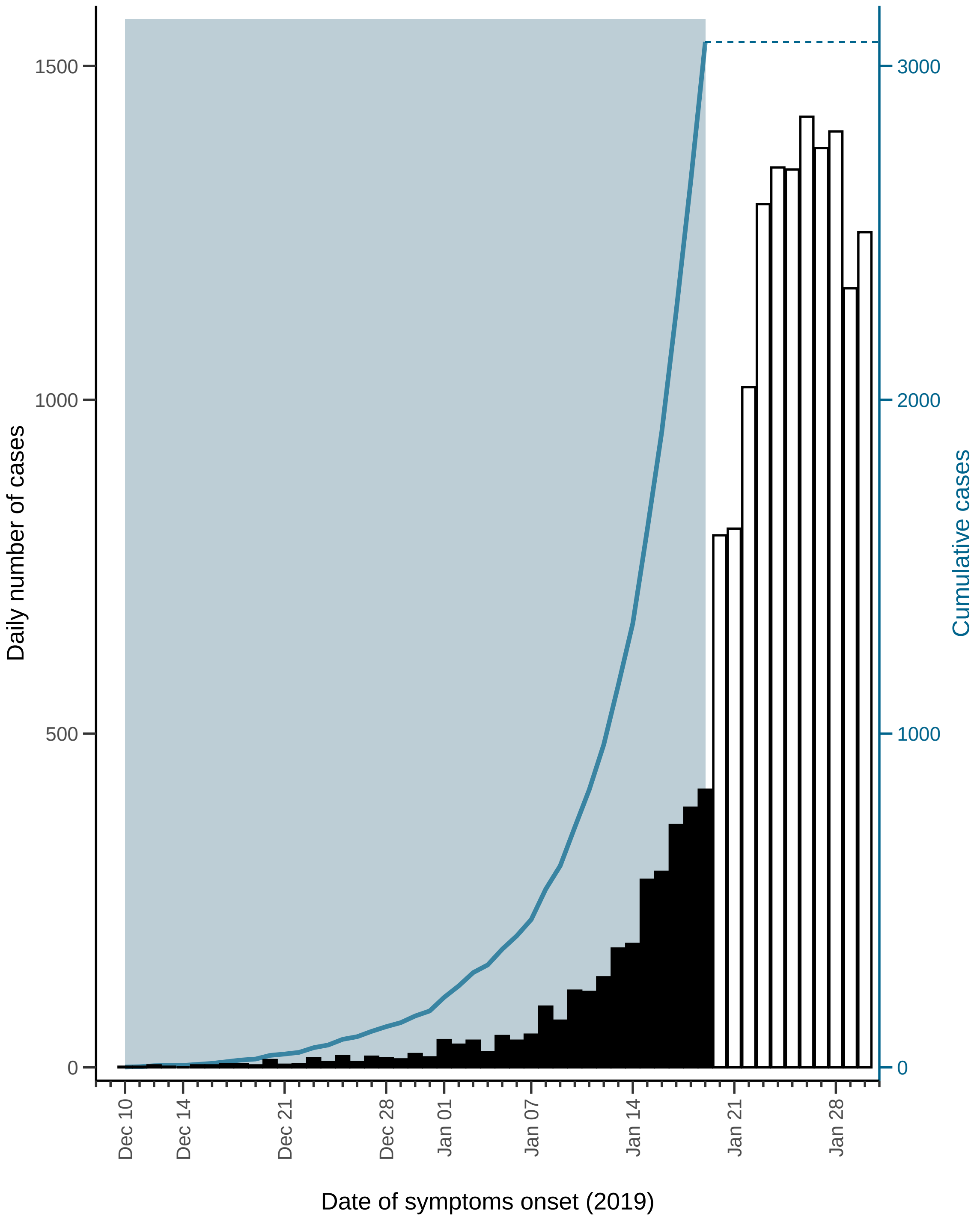

5.2 Cumulative cases

Figure H: Cumulative cases of COVID-19 in Wuhan. Accepted simulations (gray) and observed data (black).

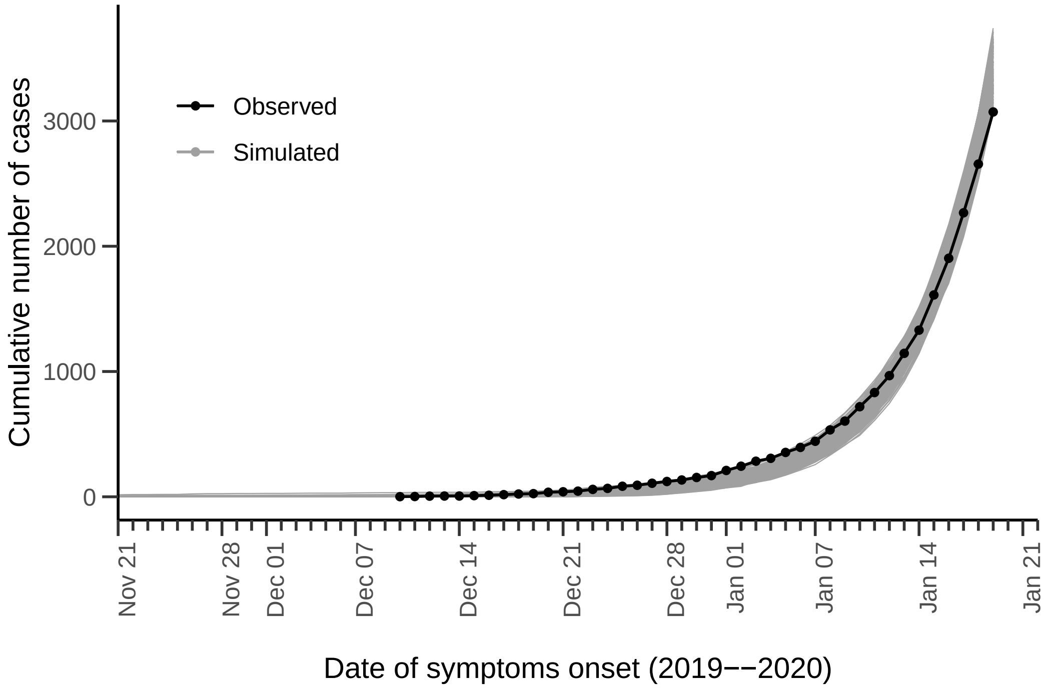

5.3 Using other COVID-19 datasets

Shorter dataset. Here, we analyze the impact of applying our model to the dataset of COVID-19 cases (cf. Figure S2) with symptoms onset before December 31, 2019, the day of the first public declaration of the Wuhan cluster [7]. Based on the 169 reported cases of COVID-19 with symptoms onset before December 31, we estimate a date of emergence by November 24 (October 29–December 04), 2019, and not earlier than September 26, 2019.

Anterior dataset. In addition, we apply our model to case data published by the WHO in 2020 [8], which were later corrected [9]. The WHO data sets used in our model are adapted from the data sets available in a GitHub repository that summarizes published epidemic curves of early COVID-19 cases [10]. Results are summarized in Table D.

Figure I: Estimates of the emergence date using truncated or outdated case datasets. Distributions of the emergence date for the COVID-19 epidemic in Wuhan, estimated using data on cases with symptoms onset by December 31, 2019 (violet, middle) and by January 19, 2020 (i.e., our main results; blue, top), as well as an outdated, later corrected dataset (red, bottom). The first cases reported in the datasets are depicted by dashed lines colored accordingly.

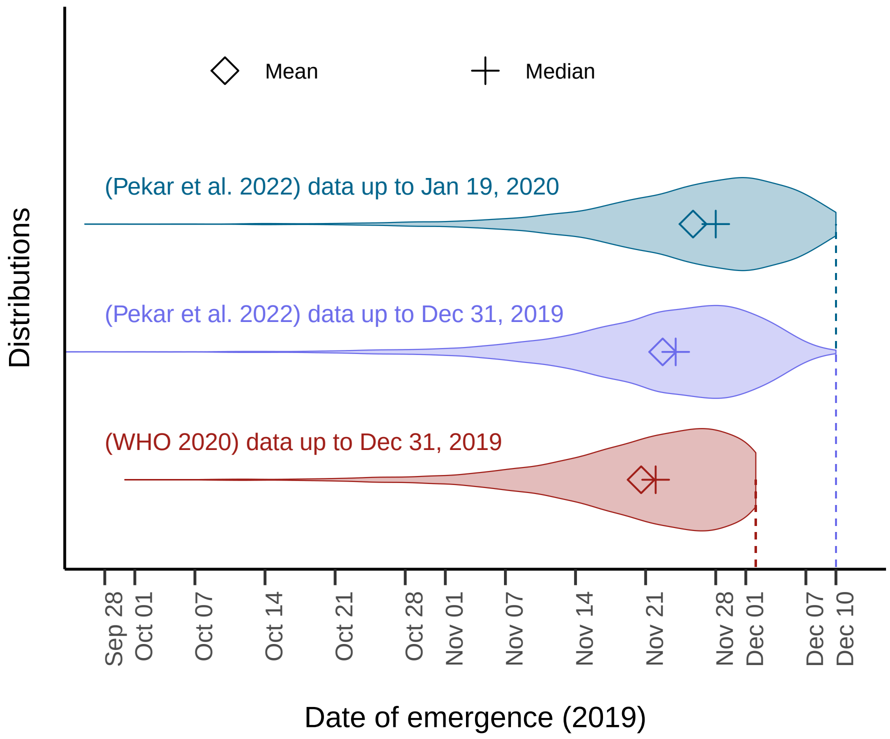

Table D: Impact of using different datasets. Results obtained using different COVID-19 cases datasets. The estimated time elapsed between the first infection to the Nth observed case yields the estimated date of outbreak emergence. In addition, we estimate the epidemic size at the date of detection of the Nth case. The proportions of detected infections are retrieved for comparison with the input epidemic parameters (cf. Table 2 of the main text). Median and 95% interpercentile ranges (i.e., values between 2.5th and the 97.5th percentiles) are shown, unless stated otherwise.

	**Case dataset**		
	**Pekar et al. 2022**[a]	**Pekar et al. 2022**	**WHO 2020**
**Observations**			
Date of Nth case	Jan 19, 2020	Dec 31, 2019	Dec 31, 2019
Date of first case	Dec 10	Dec 10	Dec 2
Number of reported cases	3 072	169	202
**Simulations**			
Emergence date (2019)	Nov 28 (Nov 2–Dec 09)	Nov 24 (Oct 29–Dec 6)	Nov 22 (Oct 28–Dec 2)
Date of earliest infection	Sep 26, 2019	Sep 24, 2019	Sep 23, 2019
Number of cases	∼3 360 (3 080–3 690)	180 (169–210)	220 (202–250)
Epidemic size[b]	∼63 400 (57 300–69 900)	∼3 500 (2 680–4 240)	∼4 160 (3 280–5 000)
Proportion of detected infections	5.31% (5.06%–5.57%)	5.3 (4.52–6.66)	5.32 (4.55–6.49)

[a] Dataset used in our baseline analyses.
[b] At day of infection of the Nth case.

6 Pseudo-algorithm

The code was built in Julia and is available at a public Github repository:
https://github.com/sjijon/estimate-emergence-from-data.

Algorithm A Estimate the delay between the first infection and the Nth case.
Abbreviations: dist. = distribution.

Require: Time-series of the N first observed cases, $\{d_i^{\text{obs}}\}_{i=1,\ldots,K'}$
Context-specific epidemiological parameters (Distributions)
Lower bound for the number of infections ($I_{\min}$)
Upper bound for the running time ($t_{\max}$)
Number of simulations (num_sims)
Error tolerance (δ_{tol})
Ensure: Time elapsed between first infection and N^{th} observed case
The simulated underlying epidemic (SimEpi)
The simulated cases (SimCases)

procedure READ DATA ▷ Returns ObsCases
    Read available data on cases
    Retrieve the number of observed cases, N, and their dates of occurence.
end procedure

while sim_num ≤ num_sims **do**

    procedure TRANSMISSION MODEL ▷ Returns SimEpi
        Initialize infections $I(t_0) = 1$
        while $t < t_{\max}$ **do**
            Draw the number of secondary infections ▷ Negative binomial dist.
            if offspring was generated **then**
                Compute the time of those secondary infections, $\{t_i\}_{i=1,2,\ldots}$ ▷ Gamma dist.
                Update number of infected at time of infection, $I(t)$
            end if
            Increase time step
        end while
        Compute daily number of infections, $I(d_k), k = 1, 2, \ldots$
    end procedure

▷ ...Continues on the next page.

Algorithm A (continuation)

if $I \geq I_{\min}$ **then**
procedure DETECTION MODEL ▷ Returns SimCases
k=1
for $d_k, k = 1, 2, \ldots$ **do**
Draw the daily number of detected infections among $I(d_k)$ ▷ Binomial dist.
Compute detection time for each individual, $\{\tau_i\}_{i=1,\ldots,M}$ ▷ Gamma dist.
Increase the daily number of cases, $Y(d_j)$
if N^{th} case detected **then**
Keep only the detections occurring on the same day as N-th case
Exit loop
end if
k=k+1
end for
end procedure
end if
Identify the epidemic size at the day where the Nth case was infected

if $N_{\text{cases}} \geq N$ **then**
procedure COMPARE SIMCASES AND OBSCASES
Condition C1: $d_1 \geq t_1$
Condition C2: $|Y^{\text{obs}}(d_k) - Y^{\text{sim}}(d_k)| \leq \delta'_{\text{tol}} N, \quad \forall k = K, K-1, K-2, \ldots$
if Conditions C1–C2 hold **then**
sim_num =+ 1 ▷ Select simulation
end if
end procedure
end if

end while

References

[1] David L. Roberts, Jeremy S. Rossman, and Ivan Jarić. Dating first cases of COVID-19. *PLOS Pathogens*, 17(6):e1009620, June 2021. ISSN 1553-7374. doi: 10.1371/journal.ppat.1009620.

[2] Chaolin Huang, Yeming Wang, Xingwang Li, Lili Ren, Jianping Zhao, Yi Hu, Li Zhang, Guohui Fan, Jiuyang Xu, Xiaoying Gu, et al. Clinical features of patients infected with 2019 novel coronavirus in wuhan, china. *The lancet*, 395(10223):497–506, 2020. doi: https://doi.org/10.1016/S0140-6736(20)30183-5. URL `https://www.thelancet.com/journals/lancet/article/PIIS0140-6736(20)30183-5/fulltext`.

[3] Peter Czuppon, Emmanuel Schertzer, François Blanquart, and Florence Débarre. The stochastic dynamics of early epidemics: probability of establishment, initial growth rate, and infection cluster size at first detection. *Journal of The Royal Society Interface*, 18(184):20210575, November 2021. ISSN 1742-5662. doi: 10.1098/rsif.2021.0575.

[4] Verity Hill, Louis Du Plessis, Thomas P Peacock, Dinesh Aggarwal, Rachel Colquhoun, Alesandro M Carabelli, Nicholas Ellaby, Eileen Gallagher, Natalie Groves, Ben Jackson, J T McCrone, Áine O'Toole, Anna Price, Theo Sanderson, Emily Scher, Joel Southgate, Erik Volz, Wendy S Barclay, Jeffrey C Barrett, Meera Chand, Thomas Connor, Ian Goodfellow, Ravindra K Gupta, Ewan M Harrison, Nicholas Loman, Richard Myers, David L Robertson, Oliver G Pybus, and Andrew Rambaut. The Origins and Molecular Evolution of SARS-CoV-2 Lineage B.1.1.7 in the UK. *Virus Evolution*, page veac080, August 2022. ISSN 2057-1577. doi: 10.1093/ve/veac080.

[5] Jonathan Pekar, Michael Worobey, Niema Moshiri, Konrad Scheffler, and Joel O. Wertheim. Timing the SARS-CoV-2 index case in Hubei province. *Science*, 372(6540):412–417, April 2021. ISSN 0036-8075, 1095-9203. doi: 10.1126/science.abf8003.

[6] Shruti Khare, Céline Gurry, Lucas Freitas, Mark B Schultz, Gunter Bach, Amadou Diallo, Nancy Akite, Joses Ho, Raphael TC Lee, Winston Yeo, Gisaid Core Curation Team, Sebastian Maurer-Stroh, GISAID Global Data Science Initiative (GISAID), Munich, Germany, Bioinformatics Institute, Agency for Science Technology and Research, Singapore, Oswaldo Cruz Foundation (FIOCRUZ), Rio de Janeiro, Brazil, Institut Pasteur de Dakar, Dakar, Senegal, National Institutes of Biotechnology Malaysia, Selangor, Malaysia, Smorodintsev Research Institute of Influenza, St. Petersburg, Russia, Genome Institute of Singapore, Agency for Science Technology and Research, Singapore, China National GeneBank, Shenzhen, China, A*STAR Infectious Disease Labs (ID Labs), Singapore, National Public Health Laboratory, National Centre for Infectious Diseases, Ministry of Health, Singapore, and Department of Biological Sciences, National University of Singapore, Singapore. GISAID's Role in Pandemic Response. *China CDC Weekly*, 3 (49):1049–1051, 2021. ISSN 2096-7071. doi: 10.46234/ccdcw2021.255.

[7] WHO. Listings of WHO's response to COVID-19, June 2020. URL `https://www.who.int/news/item/29-06-2020-covidtimeline`. [Accessed: Nov 25, 2022].

[8] The Novel Coronavirus Pneumonia Emergency Response Epidemiology Team. The epidemiological characteristics of an outbreak of 2019 novel coronavirus diseases (covid-19) – china. *China CDC Weekly*, 2:113, 2020. ISSN 2096-7071. doi: 10.46234/ccdcw2020.032.

[9] Michael Worobey. Dissecting the early COVID-19 cases in Wuhan. *Science*, 374(6572):1202–1204, December 2021. doi: 10.1126/science.abm4454.

[10] Florence Débarre. Wuhan December 2019 epi-curves, 2022. URL `https://github.com/flodebarre/covid_firstCases`. [Accessed on: December 15, 2022].
