# Supplementary material for: Using early detection data to estimate the date of emergence of an epidemic outbreak
Source: PLoS Comput Biol. 2024 Mar 8;20(3):e1011934. doi: 10.1371/journal.pcbi.1011934 (PMC10954163; doi:10.1371/journal.pcbi.1011934)
Supplement: Attachment — Submitted filename: v2_jijon_et_al_plos_comp_bio_2023.pdf [file pcbi.1011934.s003.pdf]

Referee report on *Using early detection data to estimate the date of emergence of an epidemic outbreak*

by S. Jijón, P. Czuppon, F. Blanquart and F. Débarre

1 Summary

In this study, the authors introduce a novel approach for recovering information on emerging diseases, relying on time series of early reported cases rather than genomic data. To accomplish this, they assume that the disease dynamics and detection can be modelled by a general branching process allowing for super-spreading events. This modelling framework was previously introduced in a paper by three of the authors [10], and has already been successfully applied to recover the date of emergence of an epidemic outbreak as well as the epidemic size at the time of first detection using the date of the first reported case.

The methodology used to perform inference is based on (but different from) Approximate Bayesian Computation inference methods. Specifically, a large number of simulations are generated, and only those that closely match the observed data (in the sense of a specific metric described in the article) are kept. These simulations provide an approximate posterior distribution of the actual date of emergence of the epidemic outbreak and the epidemic size at the time of the last reported case in the dataset. As this methodology enables the use of time series of early reported cases, rather than just the date of the first reported case, it represent a significant extension of the approach used in [10].

To evaluate the validity of the inference framework, the authors use two COVID-19 datasets of genomic sequences: early reported cases of the Alpha variant in the UK, and early (often retrospectively) reported COVID-19 cases in Wuhan. These datasets were previously studied in [10] or other studies based on genomic data, enabling the authors to compare their results with previous findings and assess the quality of the estimation. A sensitivity analysis was also conducted to evaluate the dependence of the estimation on the input values of the model parameters.

2 Evaluation

This article presents an innovative approach to estimate the date of emergence of an epidemic outbreak, without the need for genomic data. The paper is very well written and the methodology used is very clearly described, making it accessible to a wide readership. I believe this approach has the potential to be applicable to a wide range of diseases.

While I found the article to be well-written and informative, both datasets analysed in the study were previously studied using other approaches, and the results obtained using the new framework are described as similar to those obtained with previous methods. I understand that there are only a limited amount of available datasets, however I believe that the article should include a more in-depth discussion of whether and how the new approach improves upon previous results, in order to be suitable for publication in *PLOS Computational Biology.*

Additionally, the approach used in this paper is a substantial extension of the methodology used in [10]. In particular, I expect it to be significantly more computationally intensive. However, the estimation results for the Alpha variant dataset are described as only slightly improved compared to those obtained in [10], which suggests that the approach used in [10] already captures most of the relevant information and may be sufficient for most applications. Given that the methodology introduced in this article is an (arguably non-straightforward) extension of the one in [10], I believe that the article should provide more detailed evidence demonstrating how this new approach leads to significantly improved estimates compared to [10].

3 Comments and suggestions

The following is a list of comments and suggestions that the authors may wish to include in a revised version of the paper. Most of these are minor comments and suggestions, except for the comments indicated in italics, which correspond to more significant issues.

1. L.54-55: "infection times occurring earlier than the tMRCA can be estimated thanks to mathematical models" To me, this implies that there is no mathematical model behind tMRCA approaches, which is not the case. Did you mean that it is possible to use population dynamics models rather than population genetics processes ?

2. P.5, BEGINNING OF 2.1: You may want to define what you mean by "case" here instead of in Section 4.1

3. L.75: Did you mean "in particular" rather than "namely" ?

4. L.109-110: "not earlier" Do you mean that in none of the 5000 simulations had the first infection occurred before June 12th 2020 ?

5. Can you explain the motivations behind changing the distribution of the number of secondary cases compared to what was done in [10]?

6. L.118-120 AND L.195-196: *If I understand correctly, the estimates you obtain in this first application are only slightly improved compared to the ones obtained with the original method from [10]. However, as your new method uses the first N cases rather than the first case, I expect it to be significantly more computationally intensive. I think this is something that should be discussed in more details in the article.*

7. L.126-127: What is the difference between your model when $N = 1$ and the updated version of the model from [10] ?

8. L.122 AND L.146: "date of infection of the N-th case" Did you mean the date at which the N-th case is reported (as mentioned earlier on L.84-86).

9. TABLE 1: You may want to recall the value of N somewhere in the caption or in the table.

10. L.158-159: This statement is unclear for somebody who has not read the *Methods* section already.

11. L.173-175: Doesn't this potentially imply that the model should include parameter values changing over time to model the data accurately ?

12. L.202-203: "the novelty of these findings rests on using exclusively a population dynamics approach" However, your approach relies on the previous knowledge of several model parameters How were these parameters estimated ?

13. L.246-251: *One of the limits of your method is that it cannot be used in the case of multiple introductions. However, one of the references you cite ([1]) indicates that it is likely to be the case for the dataset of early COVID-19 cases in Wuhan.*

14. L.281-286: *I do not understand why you sample both a binomial distribution for the number of observed cases and a time from infection to detection for each case $j = 1,...,N$. Was τ_j supposed to be a time from infection to detectability instead, and in that case j is not bounded by N ?*

15. If I understood correctly, in this model, individuals stay infected and infect individuals forever. I believe you should comment on this arguably strong assumption in the *Discussion* section.

16. L.317-318: "the actual rejection criterion is actually slightly more complex than described" In particular, you may want to mention here that it relies on the whole time series of observed cases.
