# Supplementary material for: Using early detection data to estimate the date of emergence of an epidemic outbreak
Source: PLoS Comput Biol. 2024 Mar 8;20(3):e1011934. doi: 10.1371/journal.pcbi.1011934 (PMC10954163; doi:10.1371/journal.pcbi.1011934)
Supplement: Attachment — Submitted filename: Response.pdf [file pcbi.1011934.s004.pdf]

Response to the reviewers

Using early detection data to estimate the date of emergence of an epidemic outbreak

Jijón, S.[1], Czuppon, P.[2], Blanquart, F.[3] and Débarre, F.[1]

Please find attached a revised version of our manuscript. Following comments by the reviewers, we made the following major changes. i) We generated synthetic data and tested our framework on it to validate the approach. ii) We compared our optimized approach to a computationally more intensive but more classical Approximate Bayesian Computation (ABC) approach, and found similar results. iii) We revised our rejection criteria, and simplified them. iv) We added sensitivity analyses, changing the values of input parameters, but also changing the datasets used. Our findings were robust to these changes.

Below, we retyped the comments and suggestions of the reviewers, and broke them into key points (highlighted in boldface and italics). Our answers to each point immediately follow. The corresponding changes are cited in our answers below, and tracked in the main text. Other minor changes were made for clarification or consistency, and are also highlighted in the text.

NB: Reference numbering changed. Ref [10] was Czuppon et al. (2021) and Ref. [11] was Hill et al. (2022).

Reviewer #1

Jijón and colleagues present an approach for estimating the true emergence date of an infectious disease outbreak using reported case count data. They apply their approach, which involves fitting a branching process epidemiological model to the data using an inference method similar to Approximate Bayesian Computation, to two case studies: the very beginning of the COVID-19 pandemic, and the emergence of the Alpha SARS-CoV-2 variant in the UK. Overall, the subject matter is interesting, and I believe the study is a useful contribution to the literature.

We thank Reviewer #1 for their careful reading and thorough comments, as well as acknowledging the contribution of our work.

I have two main recommendations for improving the manuscript, as well as some more specific comments:

Main points:

1. More should be done to detail the advance provided by this work compared to previous studies. In particular, the authors present their approach as an extension of that in Ref. [10] (in which the emergence date was estimated based on the date of the first case only), but it would be useful

to provide some context of whether this was a straightforward extension or required substantial methodological innovation. A more detailed discussion of the advantages and disadvantages of the approach here compared to studies using genomic data (or other approaches) would also improve the narrative.

As noted by the reviewer, this work follows our previous work published in Czuppon et al. (2021), in which only the first case was used. We now explain the rationale for extending this work in the Introduction, around line 76.

This was not a straightforward extension; it required to revisit in particular the definition of rejection criteria for the simulations, and we had to write a completely new code for the simulations, since the previous methodology was completely dependent on the fact that there was just one detected case. In particular, the analytical results that we had obtained in Czuppon et al. (2021) are unfortunately now out of reach when considering $N > 1$ cases. Additionally, we tested our framework on synthetic data (main text around line 119 and Appendix 2.3 and Figure S6), and conducted more sensitivity analyses (Appendix 2.4).

Finally, we added a discussion of the value of other approaches in the introduction, around (before) line 70 and in the Discussion around line 257. Our work does not intend to replace nor outperform studies with genomic data. Indeed, our findings fall within the same ranges than those found in such studies. Hence, our work can help supporting previous findings relying on different methodologies.

An additional contribution of this work, from the methodological point of view, is its flexible nature. Our numerical implementation was conceived and built with this in mind, allowing to study different epidemiological contexts (cf. the public repository of our code: https://github.com/sjijon/estimate-emergence-from-data.), i.e. not limited to COVID-19.

2. Given the somewhat non-standard nature of the inference method used here, I recommend conducting a simulation study using synthetic case data to verify the validity of the inference approach. This should also demonstrate the advantage of using data beyond the first case when estimating the emergence date. In fact, it may be interesting (but not essential) to explore how the precision of emergence date estimates improves when using more data (particularly in light of the discussion about transmission parameters changing over time suggesting it may not in reality be optimal to use all available data).

To address this comment, we re-implemented our analysis as a more, but less computationally optimized, standard ABC. As shown in an updated Figure 3: the results are almost the same.

As mentioned in the reply to the previous comment, we also tested our method on simulated data (main text around line 119 and Appendix 2.3). We found that we were able to recover the date of the first infection (with a 2–3 day delay).

We were also able to explore the effect of the number of cases that are used for the estimation. We found that adding more cases widens the confidence intervals, and that the estimation was already rather accurate with just one case. However, these analyses were conducted on *ideal* data, in that we draw datasets from fixed distributions. Real data are not that clean, may include biases, may miss critical early cases, so we still think it is valuable to include more cases for the estimates to be robust. We revised language in the manuscript to reflect this finding.

Further comments:

1. Lines 34-52: it would be useful to explain what the tMRCA is (and why it may not be the same as the emergence date), as well as giving more context to what you mean by "adapting a technique used in conservation science" (is it possible to broadly classify what type of model was used in Ref. [4]?). One idea for providing more necessary explanation while simplifying the narrative of this paragraph may be to summarise details of specific previous studies in a table (perhaps including studies estimating emergence dates for outbreaks of pathogens other than SARS-CoV-2).

We added details on tMRCA in the Introduction section, in particular by adding a schematic as new Figure 1. We added a clarification on Ref. [4] around line 43.

2. Lines 57-59 ("In particular... epidemiology"): some references would be nice here (for example, to the large body of work considering the probability of a small number of initial cases leading to a major outbreak occurring).

We have added various references to the large body of stochastic epidemiological modeling literature, around line 68.

3. Lines 60-61: here, and similarly throughout the manuscript, you refer to estimation of the "first infection leading to a sustained epidemic" (e.g. lines 91-93). This could give the impression that you deal directly with the possibility that early cases in the data do not belong to the main outbreak under consideration (which I do not think is true, given the calibration condition for simulated outbreaks that the first simulated infection pre-dates the first observed case). Please could you clarify and rephrase accordingly throughout.

By "first infection leading to a sustained epidemic", we mean that we do not deal with transmission trees that go extinct nor, as a consequence, with data that is not contained in the transmission tree. We added a diagram, now Figure 1, to illustrate this (cf. further comment 1 above): our framework does not account for the "Unsuccessful spillovers" depicted in Figure 1. We also added to the Discussion section a limitation of our study regarding the unobserved aborted trees, around line 291.

4. Lines 60-65: this paragraph needs more detail. Specifically, I would briefly outline how extend Ref. [10], as well as adding in a sentence at the end summarising what you do with your approach.

We revised the paragraph; please refer to our response to comment 1 above.

5. Lines 126-128: related to main point 1 above, it is currently unclear how the model with $N = 1$ is any different from using the approach in Ref. [10] (given that the approach here is presented as an extension of that in [10] to $N > 1$).

The simulation code is different, but we recover results obtained in Czuppon et al. (2021) when we set $N = 1$. We added a clarification around line 164.

6. Lines 144-145: I think the phrase "remarkably close" may be an exaggeration. You could perhaps instead note that your estimates appear to be consistent with [1] but give a tighter bound.

We rephrased our statement for nuance, now around line 180 (but we were pleasantly surprised by how well the results matched in spite of very different methodologies!).

7. Sensitivity analyses: consider adding an additional analysis varying the time from infection to detection, as intuitively this would seem to be an important driver of the results.

We ran additional sensitivity analyses by varying the time from infection to detection. In summary and as expected, we found that decreasing the shape parameter of the Gamma distribution, θ_τ, reduces the time between the first infection and the N-th case. These results are now found around line 191 of the manuscript, shown in panels G and H of Figure 4 and in table S3 of the Appendix.

8. Lines 154-156 ("In addition…section"): I'm a little confused by this sentence, and would suggest adding more explanation (or deleting the sentence).

We rephrased for clarification of the effects of varying R, the mean number of secondary infections; now around line 197.

9. Lines 202-203 ("To the…subject"): can you say why the novelty of using exclusively a population-dynamics approach may be an advantage? Is it simply that genomic data may not always be available?

Yes, but also that there is value in approaching a question using different methodologies: this provides better confidence in the estimated values (line 70). We rephrased the sentence (now around line 233). In addition, in response to previous similar comments, we discussed further the contribution of our work, from the methodological perspective, around line 257.

10. Lines 216-220: can you add anything here about why quantifying the time to detection is important in these contexts?

We added elements around line 265.

11. Lines 258-263: this paragraph arguably suggests that your approach may not be as good as that of Pekar et al. (since lines 36-38 suggest their work already combined a population-level epidemiological model and genomic data), and that there may not be much advantage to the novelty of not using genomic data (cf. lines 202-203). Perhaps the key points of this paragraph could be included in a more detailed discussion of the relative differences/advantages/disadvantages of this study compared to previous work (see main point 1 above).

This was addressed in reply to main point 1 and comment 9.

12. I would recommend adding a final paragraph at the end of the Discussion summarising the main conclusions of the study.

We added a paragraph at the end of the Discussion section, as advised; see around line 312.

13. Lines 281-282: "per-day number of observed cases" could easily be misinterpreted as referring to the number of cases detected on the current day (whereas my understanding is that you are actually referring to the number of individuals, infected on the current day, who are later detected). I would rephrase.

We thank the reviewer for raising this issue. We revisited our definitions of the per-day number of observed cases to avoid confusion (now line 332).

14. Lines 294-296: I would explicitly note the (apparent) assumption that the generation time is independent of the infector's time from infection to detection. This is common and acceptable, but since a number of recent studies have noted this may not be realistic for COVID-19 and/or have relaxed this assumption, it's worth being clear about this.

We added an explicit note on independence, now around line 337.

15. Lines 298-299 ("In our...continuous scale"): I'm a little confused here (what's actually being discretised by 0.1 days?). Some more detail would be useful.

We rephrased to avoid confusion, now line 343.

16. Lines 293-296: is there potential for (slight) bias in your inference from always terminating simulations so that at least N cases have occurred (and presumably usually strictly more than N) by the end of the simulation, when the data specify exactly N cases at the corresponding time? Please clarify (for example, why choose this approach instead of matching the simulation date on which the cumulative number of cases is nearest to N to the actual date when the cumulative number of cases equals N?).

We agree with Reviewer #1 in that selecting simulations where at least N cases are observed may yield a bias. However, this choice is coherent with our assumption that a sustained epidemic is ongoing by the time data on N observations are available. In the case of also selecting simulations with values of M near but below N, because we are dealing with an exponential growth, the difference in the estimated dates, if any, would be small (probably of ~ 1 day).

17. Equation (4): I'm not sure this equation quite makes sense as stated (should the summand just be 1?).

We thank the reviewer for spotting the incorrect definition. We corrected the text and the equation (line 350).

18. Caption to Figure 4: presumably ${y_j}_1^m$ is the set of cases detected up to and including day d_k, not just on d_k itself? Also, is Y_k here the same as $Y(d_k)$ in equation (4)?

We use y_j with $j = 1,\ldots,M \geq N$ to denote the jth detected infection, and Y_k, with $k = 1,\ldots,K$ to denote the number of detected infections up to day k. We edited the caption of Figure 4 to avoid confusion.Also, we previously noted here the same as $Y(d_k) = Y_k$ to ease notation, but we now use Y_k exclusively (lines 329–330).

19. Lines 324-337: is it possible to (at least briefly) justify the choices of constraints? For example, it is not obvious why you penalize faster simulations more than slower ones in (C3), and why you use a max norm and cumulative case counts in (C4).

Following this remark, we revised and vastly simplified the conditions; see Methods line 394 and following.

Condition C2 (formerly C4) allows us to compare the cumulative number of detections, daily (which could be redefined to compare the number of per-day new detections). The max norm is applied to evaluate the highest jump in the number of new detected infections, relative to the total observed

number N, in order to penalize too large jumps.

20. Line 332: possible typo (should "we" be deleted?).

We thank the reviewer for spotting the typo. We corrected the sentence.

21. Table 2: where possible, I recommend citing the original studies in which the parameter value estimates were obtained.

While stating that we use the parameters as in Czuppon et al. (2021), we added the original studies to Table 2, as suggested.

Reviewer #2

2 Evaluation

This article presents an innovative approach to estimate the date of emergence of an epidemic outbreak, without the need for genomic data. The paper is very well written and the methodology used is very clearly described, making it accessible to a wide readership. I believe this approach has the potential to be applicable to a wide range of diseases.

While I found the article to be well-written and informative, both datasets analysed in the study were previously studied using other approaches, and the results obtained using the new framework are described as similar to those obtained with previous methods. I understand that there are only a limited amount of available datasets, however I believe that the article should include a more in-depth discussion of whether and how the new approach improves upon previous results, in order to be suitable for publication in PLOS Computational Biology.

Additionally, the approach used in this paper is a substantial extension of the methodology used in [10]. In particular, I expect it to be significantly more computationally intensive. However, the estimation results for the Alpha variant dataset are described as only slightly improved compared to those obtained in [10], which suggests that the approach used in [10] already captures most of the relevant information and may be sufficient for most applications. Given that the methodology introduced in this article is an (arguably non-straightforward) extension of the one in [10], I believe that the article should provide more detailed evidence demonstrating how this new approach leads to significantly improved estimates compared to [10].

We thank Reviewer #2 for the assessment of our manuscript and the feedback, as well as the acknowledgment of our contribution to the existing literature.

The issue of the discussion of our methodological contribution was raised by other reviewers, too. We added further details on the extension of the model in Czuppon et al. (2021) in the Methods section, as well as a further discussion on the improvements and limitations this extension offers. Please refer to our response to comment 1 of Reviewer #1 for a detailed response and the number of lines of the manuscript were the modifications were made.

We would like to however stress that our study does not intend to necessarily improve the estimations available in the literature; rather, we believe that one of our contributions is to offer a rather simple methodology to estimate the date of first infection using population-dynamics data instead of genomic data, and to provide methodologically independent confirmation of previously found estimates.

3 Comments and suggestions

The following is a list of comments and suggestions that the authors may wish to include in a revised version of the paper. Most of these are minor comments and suggestions, except for the comments indicated in italics, which correspond to more significant issues.

1. L.54-55: "infection times occurring earlier than the tMRCA can be estimated thanks to mathematical models" To me, this implies that there is no mathematical model behind tMRCA ap-

proaches, which is not the case. Did you mean that it is possible to use population dynamics models rather than population genetics processes ?

We rephrased to avoid confusion; see around line 61.

2. P.5, beginning of 2.1: You may want to define what you mean by "case" here instead of in Section 4.1

We added the definition of a case as suggested (line 91).

3. L.75: Did you mean "in particular" rather than "namely" ?

We corrected the sentence as suggested (see line 98).

4. L.109-110: "not earlier" Do you mean that in none of the 5000 simulations had the first infection occurred before June 12th 2020 ?

Indeed; we use "not earlier" when we give the minimum estimate for all 5 000 simulations. We now explain this in the main text, around line 142.

5. Can you explain the motivations behind changing the distribution of the number of secondary cases compared to what was done in [10]?

We used a negative binomial distribution to account for super-spreading. This is mentioned around line 329 of the manuscript (Methods section). Please note that the main results in Czuppon et al. (2021) result from using a Poisson distribution. However, in our manuscript, we compare our results to those obtained from running the model of Czuppon et al. (2021) using a negative binomial distribution —and a mean number of secondary infections as in Hill et al. (2022).

6. L.118-120 and L.195-196: If I understand correctly, the estimates you obtain in this first application are only slightly improved compared to the ones obtained with the original method from [10]. However, as your new method uses the first N cases rather than the first case, I expect it to be significantly more computationally intensive. I think this is something that should be discussed in more details in the article.

Please refer to our response to comment 1 of Reviewer #1 for a detailed response on the improvements and limitations this extension offers, as well as the number of lines of the manuscript where the modifications were made.

7. L.126-127: What is the difference between your model when $N = 1$ and the updated version of the model from [10] ?

We ran our model using $N = 1$ to test if our extended version of the model recovered the results of the updated version of the model from Czuppon et al. (2021) (updating the value of R to match that of Hill et al. (2022) for comparability). We added some details in the Results section, around line 164.

8. L.122 and L.146: "date of infection of the N-th case" Did you mean the date at which the N-th case is reported (as mentioned earlier on L.84-86).

We thank the reviewer for spotting the inconsistency. We do mean the epidemic size at the date of infection of the Nth case, to ensure a correct computation of a proportion. We corrected our reasoning in the Results section, around line 108.

9. Table 1: You may want to recall the value of N somewhere in the caption or in the table.

We added the value of N, the number of reported cases, for both epidemiological contexts in the caption of Table 2.

10. L.158-159: This statement is unclear for somebody who has not read the Methods section already.

We agree with Reviewer #2 on that some details may remain unclear before reading the Methods section. We rephrased slightly the statement, while adding a reference to the Methods section for details in order to avoid repetitiveness within the manuscript.

11. L.173-175: Doesn't this potentially imply that the model should include parameter values changing over time to model the data accurately ?

Indeed; one of the limitations of the way we constructed the model is that we couldn't take into account time-depending parameters, which we would expect to yield better estimates. This only affects our "optimized" model; a more classical ABC method could take time-dependent parameters into account. We now mention this in the Discussion, around line 299.

12. L.202-203: "the novelty of these findings rests on using exclusively a population dynamics approach" However, your approach relies on the previous knowledge of several model parameters. How were these parameters estimated ?

Indeed, our model relies on previous estimation of the model parameters, which was listed as a limitation of our study around line 286 of the Discussion section. We believe it remains outside the scope of our study to present the variety of methods to estimate such parameters; hence, we did not edit the manuscript in this regard.

13. L.246-251: One of the limits of your method is that it cannot be used in the case of multiple introductions. However, one of the references you cite ([1]) indicates that it is likely to be the case for the dataset of early COVID-19 cases in Wuhan.

We agree with Reviewer #2 in that it may be confusing to compare our results to a study that finds multiple emergence events, while our methods can not be applied in that situation. However, we believe our results are still relevant because, by the time our analyses were done and the time these texts are written, there is no consensus about a multiple-event emergence of SARS-CoV-2. We however expect the effect of these close multiple introductions to be limited – and it is, in that our results are very similar to Pekar et al.'s. However, it is clear that our model could not be applied to case data on Middle East Respiratory Syndrome Coronavirus (MERS-CoV) infections, where there is a consensus on multiple zoonotic events (see for instance Dudas et al. (2018; DOI: 10.7554/eLife.31257) and Arabi et al. (2017; DOI: 10.1056/NEJMsr1408795)).

14. L.281-286: I do not understand why you sample both a binomial distribution for the number of observed cases and a time from infection to detection for each case $j = 1, ..., N$. Was τ_j supposed

to be a time from infection to detectability instead, and in that case j is not bounded by N ?

We first draw the number of detections among the infections and then the time elapsed between infection and detection, from which we determine τ_j, the time of detection of the jth case. We clarified the notation to avoid confusion (see line 337).

Please note that while N is a stopping criterion for our algorithm, in the sense that we stop the simulation at the end of the day of occurrence of the Nth detection, we still deal with all M detected cases in that day. As a consequence, j is not bounded by N but by $M \geq N$. We ensured to use subscript j for τ through the whole manuscript.

15. If I understood correctly, in this model, individuals stay infected and infect individuals forever. I believe you should comment on this arguably strong assumption in the Discussion section.

We do not model infectious individuals as being able to infect forever. Rather, for each infectious individual, we draw the number of secondary infections they produce and the generation time of these secondary infections from distributions with known parameters (estimated and available in the existing literature). While these drawn infection times can be very large, they are not infinite (the tail of the generation time distribution drops exponentially). We revisited the Methods section for clarification, see around line 339.

16. L.317-318: "the actual rejection criterion is actually slightly more complex than described" In particular, you may want to mention here that it relies on the whole time series of observed cases.

We very much revised this part of the Methods and simplified the criteria vastly. We nevertheless added a mention of whole time series, as suggested (see around line 396).

Reviewer #3

This is a well-written and interesting study on how to infer the date of first infection of an epidemic using a branching process modelling approach. I have only minor comments.

We thank reviewer #3 for reading and commenting our manuscript, as well as acknowledging the interest of our work.

1. l. 277. I am not sure whether the model takes into account super-spreading events when the generation times of infections are independently distributed (l. 284). Rather than superspreading events, wouldn't it be superspreading individuals that are taken into account?

We thank the reviewer for noticing the confusion this term may cause. With "superspreading events", we intended to refer to large numbers of secondary infections that infectious individuals may produce in a short amount of time. It is however correct that the infections are not necessarily synchronous. We changed the term all over the manuscript, removing "events", to avoid confusion.

2. l. 306-321 This is the only part of the manuscript that I have had a hard time understanding. The notion that "all simulations can actually be retained and shifted" (l. 316) was lost on me, especially since afterwards I read that in fact there is only a set of "accepted" simulations (l. 321). A more mathematical or graphical explanation of this procedure would help as there does not seem to be any mention of it in the pseudo code.

We agree with the reviewer that this sentence was not clear. Indeed not all simulations are accepted and we have changed the sentence accordingly. The actual difference to classical ABC that we aim to point out in that context is that we have more precise control about the number of simulations that are underlying the approximate posterior distribution. In ABC a certain amount of simulations is initiated at every possible first date of infection and just then the rejection criterion is applied. In contrast, with our approach, we can sample one accepted epidemic after the other without specifying the first date of infection in the absolute sense because we can shift the simulations in time. We added a synthetic description of the methods around line 371, which we hope clarifies it.

Typos: l. 252 "constraint" -> constrained

l. 332 the sentence seems to be grammatically wrong.

Corrected, thank you.

Reviewer #4

This paper presents a method for estimating the origin date of an epidemic based only on a time series of cases, contingent on some fairly strong assumptions (exponential growth, all observed cases from the same spillover/introduction, constant parameters). The model is a straightforward extension of one presented in Czuppon et al (the offspring distribution is now negative binomial and the time series is used to reject simulations that deviate too far from it). These extensions make sense, but on the one example where the two methods are compared to each other the results are only slightly different, which makes it difficult to judge the impact of the extensions.

I think the model has a place, for example obtaining quick estimates of the time since a virus spillover or a new variant introduction in scenarios where sequencing data are not available or informative. It should be kept in mind that this is not a thorough investigation into the origin of the COVID-19 pandemic, nor is it a method that more accurately estimates the origin date of an epidemic than existing methods (at least that is not what is shown here). On the two examples shown here this model performs similarly to previous methods, however it is straightforward to come up with an example where previous methods that also rely on genetic data would outperform this model, such as an epidemic from multiple zoonotic spillovers, with each spillover resulting in a substantial number of cases (see Figure 1d in https://www.nature.com/articles/s41564-018-0296-2 for a hypothetical example).

We thank reviewer #4 for assessing our manuscript and giving constructive feedback, as well as acknowledging the contribution and scope of our study.

This is not a long paper, but it still has a fair amount of unnecessary repetition (e.g. most of section 2.1 is repeated elsewhere and I think Tables 1 and S1 could be merged).

Unfortunately, placing the Methods section after the Results may require to summarize some of the concepts and notations used to present and discuss our findings, which may give place to repetitive phrasing. We shortened and rephrased some of the sentences of the Results section, and refer the reader to the Methods section when necessary.

On the other hand, we would rather keep tables 1 and S1 separate, because they contain distinct information: the former summarizes our findings (i.e., estimates) while the latter summarizes the model calibration (i.e., selected simulations compared to the observations; thus placed in the Appendix).

# Major comments

1. Using the model to estimate something that is not conclusively known is not model validation. The only thing shown here is that the model recovers similar estimates to previous models, with slightly tighter credible intervals. This does not prove that this model's estimates are better in any way. It also does not prove that the model was implemented correctly or that the model is an unbiased estimator for the date of the first case, when all assumptions are met. A small simulation study would be a good way to investigate these questions. It could also be used to shed light on robustness to model violations.

We rephrased to avoid confusion and removed the term "model validation" (around line 129). We

did not intend to imply that the estimates obtained using our model are better to previous estimates, nor that the model yielded an unbiased estimator. Rather, our goal was to run our model on available data and our main results consist in recovering estimates very close to previous estimates, obtained with substantially different methods. We used the term "model validation" in the sense that we extended a previously built model and tested ours for reproduction of its results when the same conditions are met (namely, $N = 1$ in our model and Negative Binomial distribution when running the model from Czuppon et al. (2021)).

In addition, we ran additional analyses on simulated case data, using datasets of different sizes. Please refer to our answer to main comment 2 of Reviewer #1 above.

2. There is a small discussion on NPIs in Wuhan affecting estimates, but nothing about England during the emergence of Alpha. Various changes in NPIs occurred in England during October 2020 and the country went into a national lockdown on November 5th. Although the NPIs in place didn't stop Alpha from spreading it likely did have some effect on the reproductive number.

We parameterized our model as in Hill et al. (2022), where the effective reproduction number was estimated to be roughly constant over the time period considered. Additionally, since we consider data until November 11, 2020, we believe that the lockdown on November 5, 2020, only has a relatively small effect on the empirical data because the time from infection until detection is approximately of the same number of days. Therefore, the lockdown only affects the last 4–5 days of empirical data, and only to a limited extent.

3. Could the authors explain how they arrived at the fixed parameter values used here? Although the authors provide references, I think these parameter estimates are contentious and it's not clear to me why those references were chosen. In particular, the sampling probability seems like a difficult parameter to fix a priori. I think it's crucial for parameterising the model and I don't have any intuition about how one would estimate it at the start of an outbreak.

Our main objective was to apply our extended version of the model developed in Czuppon et al. (2021) to datasets of reported cases that were i) available and ii) have been used to explore the question of dating the first (unobserved) infection. For a fair comparison, we parameterized our model as close as possible to the studies performing such analyses. A sampling probability needs to be fixed for simulations to run; we used values obtained by previous studies (referenced in Table 2), but note that the proportions of detected cases that we obtain are different, but also that the impact of the input value on the results is limited (Figures 4E–4F).

4. I wonder how much the method is affected by violations to the sampling assumptions. In the model a constant sampling probability is assumed. However, we know that cases are much more likely to be ascertained on weekdays than weekends. When sequences are used as a time-series of cases fluctuations in sampling are more extreme, since sequence surveillance is not as uniform as case surveillance and it is common for the weekly sequencing capacity to be exceeded during phases of exponential growth (but this was not the case in the Alpha dataset used here). I would expect that violations to this assumption (e.g. no samples for a week or a few days with outsized contributions) could result in unnecessarily rejecting a large number of simulations and arriving at biased estimates. Moreover, if the tolerance parameters are too strict I think the model could recover fluctuations in sampling over time. Would this be the desired outcome?

For the Covid-19 example, the effect of ascertainment rate fluctuations should not be an issue, because we are working with a time series in terms of symptom onsets, which, in addition, was reconstructed retrospectively. For other examples, potential effects of such fluctuations are dampened by our use of cumulative numbers of cases in the rejection criterion C2. In the case of very large fluctuations in ascertainment rate, we would recommend using time series not in terms of detection times, but rather ones like onset dates.

5. Could the authors explain in more detail why their algorithm is not ABC? If the approximate posteriors obtained are identical I would argue that it is just a clever algorithm for performing ABC inference. I think the authors could also be more rigorous in calling the estimated distributions approximate posteriors and not just posterior distributions.

Our procedure was not exactly ABC because not all acceptance/rejection criteria were based on a distance measure. In an effort to better align our results with formal ABC, we revised the acceptance criteria in the updated version. We also compared our results with the results from a more classical (but more computationally intense) ABC procedure in Figure 3B.

# Minor comments

6. I'm pretty sure EU1 is not B.1.1778. If memory serves it's just B.1.177.

We thank the reviewer for spotting the typo. We corrected the name.

7. There may be only 406 Alpha genomes that were uploaded to GISAID by Nov 30th 2020, but there are thousands of known Alpha cases until then thanks to SGTF. See https://www.science.org/doi/full/10.1126/sci[illegible] and https://www.nature.com/articles/s41586-021-03470-x%22%22. These cases could serve as an extra robustness analysis.

SGTF data are unfortunately not useful for this kind of study because of the background noise; the signal is not specific enough. We need to be able to follow the early cases with precision. SGTF happened also outside of the Alpha variant. SGTF data are useful and reliable once the variant's proportion in the viral population is above the noise, but we are actually interested in what happened until the variant reached that proportion. This is why we could not, and therefore did not, use SGTF information.

8. Why do the authors only show the 95% IqR limits for the estimates from the new model in the figures?

Following this remark, we added IqR to the results from existing literature shown in Figures 2 and 3.

9. It doesn't look like the first case arrow in Figure 2 is lined up with the first case.

(Now Figure 3). Unfortunately, the first case doesn't seem to be shown, because of the size and scale of the figure. This is actually why we had added an arrow showing the date of the first case.

10. Table S2: The Alpha TMRCA estimate is also a phylodynamic model. It relies on the coalescent model.

We agree with the characterization; the word appears in Table S2 for Alpha tMRCA.

11. The title of Figure S6 is misleading. Are the authors suggesting that the data in Pekar et al 2022 are outdated?

We updated the title to avoid the confusion; this is now Figure S8.

12. Based on the text in the methods I think the κ_t and κ_τ parameters in Table 2 should probably be ω_t and ω_τ

We thank the reviewer for spotting the typo. We corrected the notation accordingly.

13. Line 332: There's a missing word or two in the sentence.

We rewrote this part.

14. On all the figures the date axis ticks are not uniformly spaced.

This is the case because we chose to show the same dates each month (1, 7, 14, 21, 28). Given that the number of days each month varies across the year, the number of ticks between 28 and the next 1 is uneven.

15. This is just an observation and not about this paper. It seems like the authors had to reach out to the authors of both Hill et al 2022 and Pekar et al 2022 to get the data they used in their study. It's great that they provided the data, but as both papers have been published for a while it is a little disappointing to see that the data (which are not sensitive) are still not publicly available!

We agree with the reviewer in regards with the importance of data sharing. However, we would like to stress the authors' quick response and helpful clarifications when contacted, as well as their permission to share the data they shared with us to compare our results with theirs.
