# Supplementary material for: Using early detection data to estimate the date of emergence of an epidemic outbreak
Source: PLoS Comput Biol. 2024 Mar 8;20(3):e1011934. doi: 10.1371/journal.pcbi.1011934 (PMC10954163; doi:10.1371/journal.pcbi.1011934)
Supplement: Attachment — Submitted filename: revised_version_jijon_et_al_plos_comp_bio_2023.pdf [file pcbi.1011934.s005.pdf]

Report on the revised manuscript "*Using early detection data to estimate the date of emergence of an epidemic outbreak*"

by S. Jijón, P. Czuppon, F. Blanquart and F. Débarre

The revised version of the paper addresses the different comments I had raised previously. I particularly appreciated the addition throughout the paper of an explanation of how the approach introduced in this article complements existing approaches, and I agree with the authors regarding the interest of using different methodologies to confirm previously found estimates. I also really liked the new Figure 1, which I think is really clear and a useful addition to the article.

I only have the two following relatively minor comments, which I think should be addressed before publication.

1. I think the abstract should be modified to account for the modifications made to the article. In particular, in the revised version of the paper, the model is now validated using simulated data rather than the dataset of Alpha variant infections in the UK.

2. Regarding the test of the model on simulated data, I found Figure S6 a bit confusing at first, in the sense that it seems to suggest that increasing the number of cases decreases the quality of the estimation. The results on Figure 2 seem to suggest that it is not the case, and that increasing the number of cases leads to narrower confidence intervals. I think it would be useful to include results on simulated data that clearly highlight how increasing the number of cases considered leads to better estimates. I agree with the authors that it should be the case, but to my opinion, it is important to include such results since the estimation method is an extension of one only taking into account the first reported case (and since Figure S6 seems to misleadingly suggest that only taking into account the first case leads to better estimates).
