# Supplementary material for: Using early detection data to estimate the date of emergence of an epidemic outbreak
Source: PLoS Comput Biol. 2024 Mar 8;20(3):e1011934. doi: 10.1371/journal.pcbi.1011934 (PMC10954163; doi:10.1371/journal.pcbi.1011934)
Supplement: Attachment — Submitted filename: ResponseToReviewers_R2.pdf [file pcbi.1011934.s006.pdf]

Response to the reviewers

Using early detection data to estimate the date of emergence of an epidemic outbreak
Jijón, S.[1], Czuppon, P.[2], Blanquart, F.[3] and Débarre, F.[1]

February 14, 2024

Please find below our point-by-point replies to the comments and suggestions of the reviewers. The corresponding changes are highlighted in the article.

In addition to responding to the Reviewers comments, we uploaded our code to a static repository at https://doi.org/10.5281/zenodo.10657737.

Reviewer #1

I thank the authors for fully addressing my comments.

A few very minor final comments/suggestions:

We thank the reviewer for carefully reading our response and revised manuscript.

Line 108: should "infected" be "detected"? (And similarly elsewhere – lines 159 and 185, and in table 1, penultimate row, first column, and perhaps other places).

The term "infected" is correct in the lines mentioned by the reviewer. Indeed, we keep track of the time series of infections that result in future detections. We modified slightly the sentence for clarification; see line 108.

Lines 195-198: I wonder whether (at least to some extent) the higher skewness could be an artefact of the fact that with a mean first infection date closer to the first reported case date, there are fewer possible first infection date values above the mean. If you're not sure of this, I think this sentence could just be deleted.

We followed the reviewer's suggestion and deleted the sentence.

Line 259: should "in" be "on"?

We corrected the sentence.

Figure S7: a few things don't seem quite right here: (i) I think the positions of the violins (bottom/middle/top) are stated incorrectly in the caption; (ii) the violin for the model from Czuppon

et al. (2021) here seems to be different from the corresponding one in Figure 2, but I can't see why it should be; and (iii) the current figure does not seem to support the claim in line 164. Please check and update accordingly.

i–ii) Thank you for spotting the inconsistency. We corrected the caption. The violin for the model from Czuppon et al. corresponded to their main results, as published. We updated the figure to add the violin for the results of running their method on parameters matching ours, as presented in the main text.

iii) Line 164 refers to our main results compared to the updated results from the model from Czuppon et al. updated (i.e., the two middle violin plots in fig S7). We updated the main text with a clarification.

Algorithm 1: I think lines 37 and 39 need to be updated to reflect the updated conditions.

We corrected Algorithm 1 (cf. line 38), thanks for noticing this detail.

Reviewer #2

The revised version of the paper addresses the different comments I had raised previously. I particularly appreciated the addition throughout the paper of an explanation of how the approach introduced in this article complements existing approaches, and I agree with the authors regarding the interest of using different methodologies to confirm previously found estimates. I also really liked the new Figure 1, which I think is really clear and a useful addition to the article.

I only have the two following relatively minor comments, which I think should be addressed before publication.

We thank the reviewer for reading our revised manuscript and for the appreciation of the additional material.

1. I think the abstract should be modified to account for the modifications made to the article. In particular, in the revised version of the paper, the model is now validated using simulated data rather than the dataset of Alpha variant infections in the UK.

Thank you for the remark. We corrected the abstract.

2. Regarding the test of the model on simulated data, I found Figure S6 a bit confusing at first, in the sense that it seems to suggest that increasing the number of cases decreases the quality of the estimation. The results on Figure 2 seem to suggest that it is not the case, and that increasing the number of cases leads to narrower confidence intervals. I think it would be useful to include results on simulated data that clearly highlight how increasing the number of cases considered leads to better estimates. I agree with the authors that it should be the case, but to my opinion, it is important to include such results since the estimation method is an extension of one only taking into account the first reported case (and since Figure S6 seems to misleadingly suggest that only taking into account the first case leads to better estimates).

The estimates with $N = 1$ are indeed slightly better in the simulated data, but we think the results are less reliable because they are much more sensitive to changes in the underlying data. Following other comments received at the Epidemics conference last year, we also included an additional figure for the simulated data, in which we check, for each simulated dataset, how the estimated first infection compares to the data's first infection (Figure S7).

Reviewer #4

I think the authors have sufficiently addressed my concerns. Thank you for better explaining the difference between their method and ABC. I would have liked to see a more detailed simulation study (how well can the method estimate other parameters and sensitivity to other parameters, especially the sampling probability), but given that the only real parameter of interest here is the date of the first infection, I think this is sufficient.

I have three small notes for the authors:

We thank the reviewer for reading our revised manuscript and the suggestions.

Consider using another term instead of interquantile range (perhaps percentile?). I don't see interquantile used very often and the abbreviation IQR is used for interquartile range (so a 95% IQR doesn't make sense at first read).

We now use the term "interpercentile range" and its abbreviation, IPR, to present our results.

I still find it odd that the first case doesn't show up in Fig 3A. Is it visible when zoomed into a vector graphics version of the figure?

Yes, the first case and other sole cases are visible when zooming into a vector graphics version of the figure. Unfortunately, PLOS requires TIFF versions of the figures, but the zoomable version is available on bioRxiv.

We increased the size of Figure S2 in the Appendix so sole cases are more visible and added a remark in the legend of Fig 3.

A simple hack to include genomic data on top of case data is to use the tMRCA as a rejection condition. By the assumptions of the model the first infection event in the current transmission chain is at least as old as the tMRCA. Thus, any simulation with a shorter time between the first infection and the Nth case than between the tMRCA and the Nth case should be rejected.

We thank the reviewer for their insights. It is an interesting idea. However, there is a whole distribution of possible tMRCA values, not just one, and taking into account this variability would constitute a whole separate project.
