# Supplementary material for: Using early detection data to estimate the date of emergence of an epidemic outbreak
Source: PLoS Comput Biol. 2024 Mar 8;20(3):e1011934. doi: 10.1371/journal.pcbi.1011934 (PMC10954163; doi:10.1371/journal.pcbi.1011934)
Supplement: Attachment — Submitted filename: review.docx [file pcbi.1011934.s002.docx]

Jijón and colleagues present an approach for estimating the true emergence date of an infectious disease outbreak using reported case count data. They apply their approach, which involves fitting a branching process epidemiological model to the data using an inference method similar to Approximate Bayesian Computation, to two case studies: the very beginning of the COVID-19 pandemic, and the emergence of the Alpha SARS-CoV-2 variant in the UK. Overall, the subject matter is interesting, and I believe the study is a useful contribution to the literature.

I have two main recommendations for improving the manuscript, as well as some more specific comments:

Main points:

1. More should be done to detail the advance provided by this work compared to previous studies. In particular, the authors present their approach as an extension of that in ref [10] (in which the emergence date was estimated based on the date of the first case only), but it would be useful to provide some context of whether this was a straightforward extension or required substantial methodological innovation. A more detailed discussion of the advantages and disadvantages of the approach here compared to studies using genomic data (or other approaches) would also improve the narrative.
2. Given the somewhat non-standard nature of the inference method used here, I recommend conducting a simulation study using synthetic case data to verify the validity of the inference approach. This should also demonstrate the advantage of using data beyond the first case when estimating the emergence date. In fact, it may be interesting (but not essential) to explore how the precision of emergence date estimates improves when using more data (particularly in light of the discussion about transmission parameters changing over time suggesting it may not in reality be optimal to use all available data).

Further comments:

1. Lines 34-52: it would be useful to explain what the tMRCA is (and why it may not be the same as the emergence date), as well as giving more context to what you mean by “adapting a technique used in conservation science” (is it possible to broadly classify what type of model was used in ref [4]?). One idea for providing more necessary explanation while simplifying the narrative of this paragraph may be to summarise details of specific previous studies in a table (perhaps including studies estimating emergence dates for outbreaks of pathogens other than SARS-CoV-2).
2. Lines 57-59 (“In particular…epidemiology”): some references would be nice here (for example, to the large body of work considering the probability of a small number of initial cases leading to a major outbreak occurring).
3. Lines 60-61: here, and similarly throughout the manuscript, you refer to estimation of the “first infection leading to a sustained epidemic” (e.g. lines 91-93). This could give the impression that you deal directly with the possibility that early cases in the data do not belong to the main outbreak under consideration (which I do not think is true, given the calibration condition for simulated outbreaks that the first simulated infection pre-dates the first observed case). Please could you clarify and rephrase accordingly throughout.
4. Lines 60-65: this paragraph needs more detail. Specifically, I would briefly outline how extend ref [10], as well as adding in a sentence at the end summarising what you do with your approach.
5. Lines 126-128: related to main point 1 above, it is currently unclear how the model with N=1 is any different from using the approach in ref [10] (given that the approach here is presented as an extension of that in [10] to N>1).
6. Lines 144-145: I think the phrase “remarkably close” may be an exaggeration. You could perhaps instead note that your estimates appear to be consistent with [1] but give a tighter bound.
7. Sensitivity analyses: consider adding an additional analysis varying the time from infection to detection, as intuitively this would seem to be an important driver of the results.
8. Lines 154-156 (“In addition…section”): I’m a little confused by this sentence, and would suggest adding more explanation (or deleting the sentence).
9. Lines 202-203 (“To the…subject”): can you say why the novelty of using exclusively a population-dynamics approach may be an advantage? Is it simply that genomic data may not always be available?
10. Lines 216-220: can you add anything here about *why* quantifying the time to detection is important in these contexts?
11. Lines 258-263: this paragraph arguably suggests that your approach may not be as good as that of Pekar et al. (since lines 36-38 suggest their work already combined a population-level epidemiological model and genomic data), and that there may not be much advantage to the novelty of not using genomic data (cf. lines 202-203). Perhaps the key points of this paragraph could be included in a more detailed discussion of the relative differences/advantages/disadvantages of this study compared to previous work (see main point 1 above).
12. I would recommend adding a final paragraph at the end of the Discussion summarising the main conclusions of the study.
13. Lines 281-282: “per-day number of observed cases” could easily be misinterpreted as referring to the number of cases detected on the current day (whereas my understanding is that you are actually referring to the number of individuals, infected on the current day, who are later detected). I would rephrase.
14. Lines 294-296: I would explicitly note the (apparent) assumption that the generation time is independent of the infector’s time from infection to detection. This is common and acceptable, but since a number of recent studies have noted this may not be realistic for COVID-19 and/or have relaxed this assumption, it’s worth being clear about this.
15. Lines 298-299 (“In our…continuous scale”): I’m a little confused here (what’s actually being discretised by 0.1 days?). Some more detail would be useful.
16. Lines 293-296: is there potential for (slight) bias in your inference from always terminating simulations so that at least N cases have occurred (and presumably usually strictly more than N) by the end of the simulation, when the data specify exactly N cases at the corresponding time? Please clarify (for example, why choose this approach instead of matching the simulation date on which the cumulative number of cases is nearest to N to the actual date when the cumulative number of cases equals N?).
17. Equation (4): I’m not sure this equation quite makes sense as stated (should the summand just be 1?).
18. Caption to Figure 4: presumably {y_j}_1^m is the set of cases detected up to and including day d_k, not just on d_k itself? Also, is Y_k here the same as Y(d_k) in equation (4)?
19. Lines 324-337: is it possible to (at least briefly) justify the choices of constraints? For example, it is not obvious why you penalize faster simulations more than slower ones in (C3), and why you use a max norm and cumulative case counts in (C4).
20. Line 332: possible typo (should “we” be deleted?).
21. Table 2: where possible, I recommend citing the original studies in which the parameter value estimates were obtained.
